# Research progress on astrocyte autophagy in ischemic stroke

**DOI:** 10.3389/fneur.2022.951536

**Published:** 2022-08-30

**Authors:** Pei-Wei Su, Zhe Zhai, Tong Wang, Ya-Nan Zhang, Yuan Wang, Ke Ma, Bing-Bing Han, Zhi-Chun Wu, Hua-Yun Yu, Hai-Jun Zhao, Shi-Jun Wang

**Affiliations:** ^1^College of Traditional Chinese Medicine, Shandong University of Traditional Chinese Medicine, Jinan, China; ^2^School of Nursing, Shandong University of Traditional Chinese Medicine, Jinan, China; ^3^Shandong Co-innovation Center of Classic Traditional Chinese Medicine Formula, Shandong University of Traditional Chinese Medicine, Jinan, China

**Keywords:** ischemic stroke, autophagy, astrocyte, apoptosis, drug therapy

## Abstract

Ischemic stroke is a highly disabling and potentially fatal disease. After ischemic stroke, autophagy plays a key regulatory role as an intracellular catabolic pathway for misfolded proteins and damaged organelles. Mounting evidence indicates that astrocytes are strongly linked to the occurrence and development of cerebral ischemia. In recent years, great progress has been made in the investigation of astrocyte autophagy during ischemic stroke. This article summarizes the roles and potential mechanisms of astrocyte autophagy in ischemic stroke, briefly expounds on the crosstalk of astrocyte autophagy with pathological mechanisms and its potential protective effect on neurons, and reviews astrocytic autophagy-targeted therapeutic methods for cerebral ischemia. The broader aim of the report is to provide new perspectives and strategies for the treatment of cerebral ischemia and a reference for future research on cerebral ischemia.

## Introduction

Stroke is an acute focal injury of the central nervous system initiated by vascular events. It is the main cause of disability, and the second most common cause of death worldwide (1, 2). Stroke is sub-divided into two types: hemorrhagic stroke and ischemic stroke. Ischemic stroke evoked by arterial occlusion accounts for 71% of all strokes (3). After ischemic stroke, astrocytes reportedly have a stronger ability to resist cerebral ischemic stress than neurons and play a significant neuroprotective role, holding promise as a key protector against ischemic damage (4). Notably, however, relevant pathophysiological mechanisms related to this have not been elucidated.

Autophagy is an evolutionarily conserved intracellular lysosomal degradation pathway mediated by autophagy-related genes (ATGs) (5). Under physiological conditions, autophagy serves as an intracellular catabolism regulatory mechanism that prompts cells to adapt to metabolic demands and plays an important role in development, differentiation, maturation, immunity, and disease prevention (5–7). Under pathological conditions, autophagy interferes with cell homeostasis by maintaining the bioenergy of damaged cells, clears protein aggregates and damaged organelles, and participates in the pathophysiological processes of diseases such as malignant tumors and cardiovascular and nervous system disorders (8, 9).

Accumulating evidence indicates that astrocyte autophagy regulates the survival and death of neurons through a variety of pathways and factors in the context of cerebral ischemia, reducing or aggravating cerebral ischemic injury (10–12). Therefore, astrocyte autophagy is likely to be a crucial therapeutic target in cerebral ischemia. Based on the understanding of the biological mechanism of autophagy, this report systematically expounds on the roles, molecular mechanisms, and effects of astrocyte autophagy in cerebral ischemia, and offers new ideas and strategies for the clinical treatment of cerebral ischemia.

## Molecular mechanisms of autophagy

**Figure 1:**
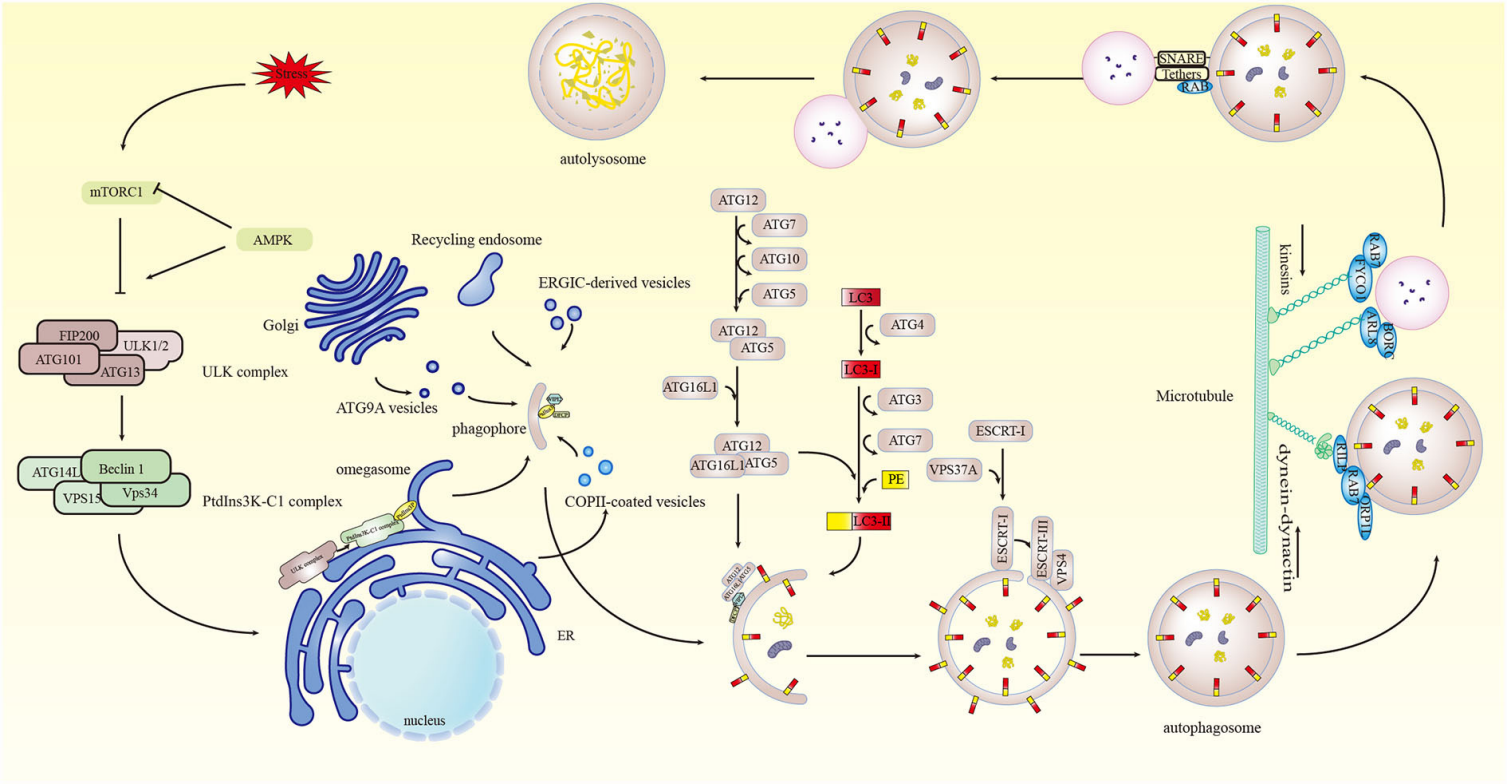
Molecular process of autophagy. Stresses stimulate mTORC1, initiating autophagy. Under the action of mTORC1 and AMPK, ULK1 in the ULK complex is activated, which in turn activates VPS34 in PtdIns3K-C1, generating PtdIns3P in the omegasome. ATG9A vesicles, recycling endosomes, and COPII-coated vesicles are involved in phagophore generation and nucleation. PtdIns3P recruits DFCP1 and WIPI2 for phagophore nucleation and expansion. The ATG12 system and the LC3 system regulate phagophore elongation, and the ESCRT mechanism regulates phagophore sealing, forming autophagosomes. Microtubule-based motor kinesins, FYCO1-RAB7 and BORC-ARL8 complexes, and dynein-dynein motor complex with RAB7, RILP, and ORP1L participate in the fusion of autophagosomes with lysosomes. SNARE complexes, tethers, and Rab proteins act synergistically in the fusion of autophagosomes and lysosomes to form autophagolysosomes.

In mammals, autophagy exhibits three forms: chaperone-mediated autophagy, microautophagy, and macroautophagy (13). Relative to the aforementioned two processes, macroautophagy is the most widespread and classical form of autophagy (14) (hereinafter referred to as autophagy), in which proteins or organelles in the cytoplasm are wrapped by double-membrane autophagosomes and fused with lysosomes to form autophagolysosomes, the contents of which are then degraded (8). The process entails the initiation of autophagy, autophagosome formation, the formation of autophagolysosomes, and the degradation of their contents (Figure 1).

### Autophagy initiation

The initiation of autophagy is controlled and affected by a series of molecules. Intracellular autophagy is induced when the body suffers adverse events such as nutrient deficiency, hypoxia, oxidative stress, pathogen infection, or endoplasmic reticulum (ER) stress (15). Initially, the nutrient sensor mammalian target of rapamycin complex 1 (mTORC1) and the energy sensor AMP-dependent protein kinase (AMPK) serve as upstream regulators of autophagy and modulate the critical autophagy-initiating kinase Unc-51-like autophagy-activating kinase 1 (ULK1), inducing autophagy (16–18). The protein kinase ULK complex is composed of ULK1/2, FIP200, ATG13, and ATG101 (19), and the autophagy-specific class III phosphatidylinositol 3-kinase complex I (PtdIns3K-C1) consisting of PIK3C3/vacuolar protein sorting 34 (VPS34), PIK3R4/VPS15, Beclin1, and ATG14L are then successively translocated and recruited to the phosphatidylinositol synthase (PIS)-rich ER subregion which consequently drives the autophagy cascade (20–22).

### Formation of autophagosomes

The formation of autophagosomes involves the processes of phagophore nucleation, elongation, and sealing. Phagophore is regenerated from small, flat cisterna and ATG9A vesicles, and recycling endosomes, COPII-coated vesicles, and the ER-Golgi intermediate compartment (ERGIC) are potentially involved in phagophore generation and nucleation (23). Phagophore nucleation and growth can be regulated by the PtdIns3P-specific recruitment proteins zinc finger FYVE-type containing 1 (ZFYVE1/DFCP1) and WD repeat domain phosphoinositide-interacting protein 2 (WIPI2), but the regulatory mechanism of phagophore nucleation remains unclear (24, 25).

Phagophore elongation is modulated by two ubiquitin-like protein-binding systems, the ATG12 system and the microtubule-associated protein 1 light chain 3 (LC3) system (26, 27). ATG12 is activated by the E1-like enzyme ATG7, then forms a thioester intermediate with ATG7 and the E2-like enzyme ATG10, then ATG12 binds to ATG5 and forms the complex ATG12-ATG5-ATG16L1 with ATG16L1 dimer (27–29). In the LC3 system, the protease ATG4 cleaves the C-terminus of LC3 to generate LC3-I, which binds to phosphatidylethanolamine (PE) and is converted to autophagosome membrane-bound LC3-II via the action of the E1-like enzyme ATG7, E2-like enzyme ATG3, and ATG12-ATG5-ATG16L1 resulting in adsorption (30, 31).

The process of phagophore sealing contributing to the formation of autophagosomes is directly regulated by the endosomal sorting complex required for transport (ESCRT) machinery (32–34). It has also been reported that the ATG-conjugated system, Rab guanosine triphosphatases, soluble N-ethylmaleimide-sensitive factor attachment protein receptors (SNAREs), sphingomyelin, and calcium are involved in the process of phagophore sealing (35, 36).

### Formation of autophagolysosomes

SNARE complexes, tethers, phosphoinositides, and Rab proteins collectively mediate the fusion of autophagosomes with lysosomes/endosomes (37, 38). The transport of autophagosomes and lysosomes via microtubules is crucial for their successful fusion (37). The homotypic fusion and vacuole protein sorting complex, a key tethering factor in the fusion process, interacts with guanosine triphosphatases and other proteins to promote cross-linking of autophagosome membranes and endosome/lysosome membranes (39). The SNARE complex, the most critical factor in the fusion process, drives the fusion of autophagosomes and lysosomes to form autophagolysosomes (40). Ultimately autophagolysosomes are degraded by lysosomes, and the contents are released into the cytoplasm generating energy and substances for cellular reorganization and homeostasis (41).

## Ischemic stroke and autophagy

**Figure 2:**
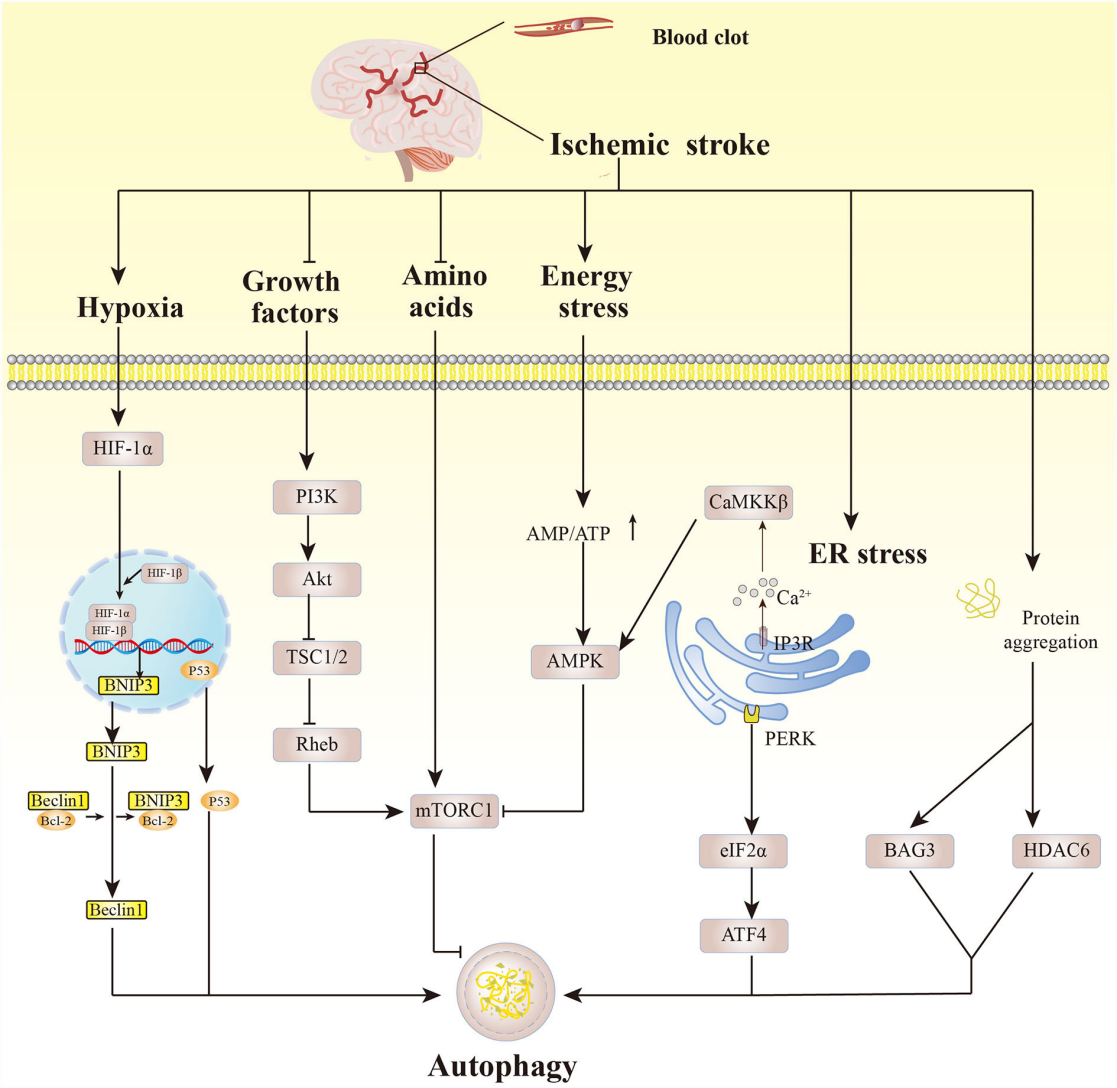
Autophagy pathways affected by pathological changes after cerebral ischemia. Hypoxic conditions activate HIF-1α which then binds to HIF-1β, and BNIP3 dissociates Beclin1 from the Beclin1/Bcl-2 complex, eliciting autophagy. p53 also induces autophagy. Growth factors that are deficient after cerebral ischemia activate PI3K, inducing Akt phosphorylation, then inhibit TSC1/2 and Rheb, activating mTORC1. Amino acid deficiency under cerebral ischemia induces autophagy by inhibiting mTORC1 activity. Similarly, energy exhaustion leads to increased AMP/ATP, and activation of AMPK inhibits the activity of mTORC1, mediating autophagy. Cerebral ischemia can trigger the release of Ca2^+^ from the inositol 1,4,5-triphosphate receptor on the ER, activating CaMKKβ, thereby activating AMPK. ER stress mediates autophagy mainly through the PERK-eIF2α-ATF4 pathway. Excessive misfolded proteins caused by ischemic injury promote autophagy by upregulating Bcl2-associated athanogene 3 and histone deacetylase 6.

Acute ischemic occlusion and decreased cerebral arterial blood flow in ischemic stroke cause insufficient oxygen supply to the brain and reduced glucose. This results in severe stress responses and a series of metabolic disorder-induced pathological changes including energy exhaustion, increased calcium ion concentration, excitotoxicity, cytokine-mediated cytotoxicity, oxidative stress, and inflammation (42). These alterations trigger the autophagic process in ischemic stroke (Figure 2).

Initially, hypoxia in brain tissues elicits enhanced activity of hypoxia-inducible factor-1 (HIF-1), which in turn regulates autophagy. B-cell lymphoma 2 (Bcl2)-interacting protein (BNIP3), the direct target gene of HIF-1α (the oxygen-regulated HIF-1 subunit), competes with Beclin1 to form the BNIP3/Bcl-2 complex and dissociates Beclin1, accelerating autophagy. Hypoxia-induced HIF-1α can stabilize p53, activating autophagy (43–45). The deficiency of glucose and growth factors after ischemia then reduces the activity of the PI3K/Akt pathway, which in turn inhibits the activity of the nutrient sensor mTORC1 and induces the autophagy pathway (46, 47). Energy is exhausted in brain cells, and the AMP/ATP ratio is greatly increased, leading to AMPK activation. AMPK has a direct role in initiating the autophagy pathway and inhibits the activity of mTORC1, boosting the autophagy process (48, 49). Ca2^+^ can be released through the inositol 1,4,5-triphosphate receptor in the ER calcium channel under ischemic conditions, and this is closely linked to ER stress (50). Cerebral ischemic injury can trigger ER stress, activating unfolded protein response (UPR). UPR core component protein kinase R-like ER kinase (PERK)-induced upregulation of eukaryotic translation initiation factor 2α (eIF2α) activates the translation of transcription factor 4 (ATF4), indirectly participating in autophagy regulation (10, 51).

After cerebral ischemia, the ubiquitin-proteasome system can degrade the misfolded proteins induced by it. When the ubiquitin-proteasome system is overloaded, however, excess misfolded proteins induce autophagy by increasing the expression of Bcl2-associated athanogene 3 and histone deacetylase 6, by which the misfolded proteins are eliminated through the autophagy-lysosomal pathway (52, 53). Previous studies have mostly focused on the induction of autophagy in brain tissues, especially neurons, through pathological changes after cerebral ischemic injury. The important role, intervention factors, and specific regulatory mechanism of glial autophagy, as well as whether there are differences between neuronal autophagy and glial autophagy, need to be further studied.

## Autophagy of astrocytes in ischemic stroke

Under physiological conditions, astrocytes in brain tissues exhibit a relatively high level of basal autophagy to maintain cellular homeostasis and function (11, 54). There are rarely autophagosomes in healthy neurons in brain tissue, indicating that neuronal basic autophagy is at a low level under normal physiological conditions (55). Knocking out core autophagy regulatory genes in neurons and astrocytes during the normal growth process of organisms leads to neurological degeneration and brain dysfunction (56–58). Thus, basic autophagy is essential for the normal functioning of neurons and astrocytes.

Ischemia-hypoxia injury deregulates autophagy, and also promotes the transformation of basic autophagy in astrocytes and neurons into induced high-level autophagy (54, 59, 60). Another study revealed that astrocytes are more sensitive than neurons, with stronger autophagic responses under the stimulation of metabolic and ischemic solutions (61). Ischemic injury after ischemia/reperfusion significantly elevates the level of autophagic flux in astrocytes, and they retain more autophagic mechanistic function than neurons (11). It is therefore essential to ascertain the specific mechanism of autophagy in damaged astrocytes in ischemic stroke. Recent reports indicate that astrocyte autophagy may have a dual effect on ischemic stroke (Table 1) and it is regulated by multiple molecules and pathways.

**Table 1:** Roles of astrocyte autophagy in experimental models of ischemic stroke.

**Animals/cell lines**	**Model**	**Time**	**Autophagy interventions**	**Roles of astrocyte autophagy in ischemic stroke**	**References**
Male SD rats	pMCAO	1–24 h	3-MA (300,600 nM)	Detrimental	(62)
Primary astrocytes from SD rats	OGD	0.5–12 h	3-MA (10 mM), Baf-A1 (1, 2, 4 μM)		
Primary astrocytes from Wistar rats	OGD	1–12 h	3-MA (10 mM), CQ (5 μM), Baf-A1 (1 μM), Compound C (20 mM), siRNAs against AMPKa1	Beneficial	(54)
C8-D1A astrocyte cell line	H/R cell model with 100 μM zinc ions	H/R 3 h/18 h	3-MA (2.5 mM)	Detrimental	(65)
Primary astrocytes from Wistar rats	OGD	1–24 h	3-MA (1 mM)	Detrimental	(67)
Primary astrocytes from SD rats	OGD/R	6/12 h	Beclin1 knockdown	Beneficial	(66)
Primary astrocytes from SD rats	OGD	0–12 h	3-MA (0.1, 0.5, 1 mM), Wortmannin (25, 50, 100 nM), ATG5 knockdown	Detrimental	(63)
Male C57BL/6 mice	tMCAO	1/24 h	circHectd1 knockdown	Detrimental	(64)
Primary astrocytes from C57BL/6J mice	OGD/R	3/0–12 h	circHectd1 knockdown		
Primary astrocytes and neurons from C57BL6 mice	neuron-astrocyte co-cultures OGD/R	0–60 min/24 h	Rapamycin (200 nM), 3-MA (10 mM), ATG5 knockdown	Beneficial	(11)
Male C57/BL6 mice	tMCAO	1/24 h	Injection with AAV-GFAP-ATG7		
Male SD rats	pMCAO	3–24 h	RIP1K knockdown	Detrimental	(124)
Primary astrocytes from SD rats	OGD	3–12 h			
Primary astrocytes from C57BL/6J mice	OGD/R	3/24 h	EGFP-fused FUS WT	Detrimental	(130)

Ref, references; SD, Sprague-Dawley; pMCAO, permanent middle cerebral artery occlusion; OGD, oxygen-glucose deprivation; 3-MA, 3-methyladenine; Baf-A1, bafilomycin A; CQ, chloroquine; siRNAs, small interfering RNAs; H/R, hypoxia/reoxygenation; OGD/R, oxygen-glucose deprivation/reoxygenation; tMCAO, transient middle cerebral artery occlusion.

### Dual roles of astrocyte autophagy

#### Detrimental role

The activation and development of autophagy can cause and aggravate the ischemic injury of astrocytes. Qin et al. (62) reported that astrocyte autophagy is activated and cellular autophagic vacuoles are increased in the cortical core region of permanent middle cerebral artery occlusion (MCAO). Oxygen-glucose deprivation (OGD) induces autophagy of astrocytes, and markedly increases the expression of the early autophagy marker protein Beclin1 and the LC3-II/LC3-I ratio (62). The addition of the autophagy inhibitor 3-methyladenine (3-MA) and bafilomycin A1 (Baf-A1) mildly but significantly inhibits the death of damaged astrocytes (62). It has also been reported that the activation of autophagy results in ischemic damage to astrocytes (63). Under OGD conditions, ATG5 and LC3-II are downregulated in ATG5-knockout astrocytes, and 3-MA and the autophagy inhibitor wortmannin repress OGD-induced astrocyte autophagy and the activation of cysteinyl aspartate-specific protease 3 (caspase-3) but increase the number of active astrocytes (63). The circular RNA HECTD1 (circHECTD1) is significantly increased in ischemic brain tissues, and knockdown of circHECTD1 restrains astrocyte autophagy and ameliorates neuronal damage and brain edema (64). The presence of excess zinc ions after ischemic stroke markedly aggravates brain injury, and 3-MA suppresses the autophagy and death of astrocytes caused by hypoxia/reoxygenation in the presence of zinc ions *in vitro* (65).

#### Protective role

Autophagy reportedly has a protective effect on astrocytes after ischemia and hypoxia. Pamenter et al. (54) reported that Beclin1 and LC3-II in astrocytes were markedly increased during 2 to 6 h of oxygen-glucose deprivation (OGD), and similar observations were reported by Qin et al. (62). Dissimilarly, 4 and 12 h of treatment of OGD-exposed astrocytes with 3-MA and the autophagy inhibitor chloroquine significantly reduced astrocyte viability, whereas Baf-A1 treatment had little effect (54). In another study, Beclin1 knockout reduced LC3-II levels, inhibited the degradation of p62 in OGD-treated astrocytes, and increased caspase-3 expression and the number of apoptotic cells (66). Intriguingly, caspase 3 cleavage levels are elevated and cell apoptosis is strengthened in astrocytes after 1 and 4 h of exposure to OGD and treatment with 3-MA, whereas astrocyte viability is reduced after 4, 8, and 24 h of exposure, and autophagy inhibits the astrocyte apoptosis elicited by OGD (67).

Multiple factors affect astrocyte autophagy. Most studies have investigated astrocyte autophagy *in vitro*, possibly due to the difficulty of quantitatively determining the amount of autophagy in astrocytes *in vivo*. Notably, the different roles of autophagy in the regulation of ischemic injury may be relevant to the discrepancies in the length of anaerobic time, presence/absence of reoxygenation, and length of reoxygenation time in reported studies, as well as differences in autophagy inhibitors and concentrations. Wang et al. (10) reported that moderate autophagy could maintain cellular homeostasis and that long-term excessive autophagy could lead to cell death due to excess cellular adaptive capacity. Therefore, astrocyte autophagy may be a “double-edged sword” in cerebral ischemia, due to different degrees of ischemic and hypoxic damage after cerebral ischemia, and varying durations of autophagy activation.

### Astrocyte autophagy-regulatory pathways

#### mTOR

mTOR is a serine/threonine kinase that regulates intracellular transcription, translation, protein degradation, and metabolism (68). The mTOR inhibitor rapamycin attenuates astrocyte proliferation and production of inflammatory cytokines following OGD/reoxygenation (OGD/R) (69). Another study indicates that rapamycin elicits the autophagy of astrocytes after OGD/R (12). However, it has also been reported that OGD triggers astrocyte autophagic flux, accompanied by an increase in the level of phosphorylated (p)-mTOR, that is, mTOR can regulate astrocyte autophagy (70).

#### AMPK

AMPK is a serine/threonine kinase that is a key regulator of the intracellular energy metabolic balance (71) and can positively regulate autophagy in astrocytes. Liver kinase B1, AMPKα1, and acetyl-CoA carboxylase are phosphorylated and upregulated when autophagy is induced in ischemic astrocytes, whereas AMPKα1 silencing and its inhibitor can downregulate the expression of astrocyte autophagy protein (54). As described above, the upstream regulators of autophagy mTORC1 and AMPK regulate the initiation of autophagy by activating ULK1 (16, 17). Another study identified the involvement of the AMPK/mTOR/ULK1 pathway in astrocyte autophagy (72). Upon elevation of autophagy levels in astrocytes after OGD exposure, p-AMPK and p-ULK1 protein levels are markedly elevated and mTOR is dephosphorylated (72).

#### Non-coding RNA-mediated autophagy

Non-coding RNAs have emerged as mediators of the autophagy pathway (73). They do not encode proteins and include microRNAs (miRNAs) that control translational functions, circular RNAs (circRNAs) that mediate miRNA functions, and long noncoding RNAs (lncRNAs) that regulate transcriptional functions (74). Upregulation or downregulation of miRNAs may induce autophagy under stress conditions (75). Zhao et al. (66) reported that miR-30d inhibited Beclin1 in astrocytes and that miR-30d antagomir expedited autophagy in OGD-treated astrocytes, which could be reversed by knocking out Beclin1 (66). CircHECTD1 is significantly elevated in brain tissues after cerebral ischemia and acts as a sponge of endogenous miR-142 to inhibit its activity, thereby upregulating the expression of TCDD inducible poly(ADP-ribose) polymerase (TIPARP) and promoting autophagy (64). Conversely, circHECTD1 knockdown reverses this process and partially alleviates ischemic injury (64). circ_0025984 and ten-eleven translocation (TET) family protein 1 (TET1) are the sponge and target of miR-143-3p, respectively, and under OGD conditions, they are downregulated in astrocytes, whereas miR-143-3p is significantly upregulated. miR-143-3p inhibitor and circ_0025984 and TET1 overexpression can dramatically reduce astrocyte apoptosis and autophagy in mice with cerebral ischemia (76). To date, there are no reports on the regulation of lncRNAs and astrocyte autophagy in cerebral ischemia.

#### Post-translational modifications

Autophagy protein activity and autophagy development are widely regulated by post-translational modifications (PTMs). PTM is a biochemical process that regulates protein activity and function (77). PTMs are well-known for their roles in autophagy, and phosphorylation and ubiquitin are two common types (78). Other types of PTM regulations such as acetylation, O-glcnacylation, and N6-methyladenosine modification reportedly play key roles in autophagy (79–82). Rahman (83) concluded that increasing O-glcnacylation did not affect astrocyte autophagy levels, and that inhibiting it promoted the autophagy of mouse cortical astrocytes. Hypoxia induces acetylation of p21 [RAC1] activated kinase 1 (PAK1), which promotes the phosphorylation and binding of ATG5 in threonine 101, thereby enhancing autophagy and promoting glioblastoma (84). Similarly, the autophagy-related gene ULK2 was found to be silenced via methylation in glioblastoma and plays an important role in astrocyte transformation *in vitro* via autophagy (85). Phosphorylation of astrocyte gap junction protein connexin-43 (Cx43) is the initiator of selective autophagic degradation of Cx43 during ischemic injury (86). Ubiquitination of Cx43 is involved in autophagic degradation, but the relationship between the two is currently unclear (86). Therefore, these types of PTMs play important roles in astrocyte autophagy, but the specific intervention modes and mechanisms involving PTMs and astrocyte autophagy after cerebral ischemia require further investigation.

### Differences between astrocytes and neurons in the autophagy pathway

The autophagy process differs in astrocytes and neurons under stress. During nutritional deprivation and metabolic stress induced by the autophagy inducer rapamycin, astrocyte autophagy is more strongly activated than neuron autophagy (87). Similarly, Moruno-Manchon et al. (88) reported that the autophagy related to sphingospkinase 1 was enhanced in astrocytes under starvation conditions, whereas neurons were not affected, and that the autophagy inducer benoxazine upregulated neuronal autophagy and sphingospkinase 1, but astrocytes were not affected. Autophagy reactions between astrocytes and neurons differ under hypoxic-ischemic conditions, and studies indicate that there may be differences in the autophagy pathway between astrocytes and neurons in cerebral ischemia.

MTOR and AMPK are classic pathways that regulate autophagy in astrocytes and neurons after cerebral ischemic injury. Regulated in development and DNA damage responses 1, TP53-induced glycolysis and apoptosis regulator, P53, and lncRNA C2dat2 are upstream of mTOR and regulate the autophagy pathway of mTOR in neurons (89–93). Similarly, Sirtuin3, LncR-AC136007.2, and α7 nicotinic acetylcholine receptor (α7nAChR) can also affect the neuronal AMPK autophagy pathway (94–96). However, it is not known whether these or other regulatory factors affect the autophagy pathway of astrocytes. The classical autophagy regulatory pathway PI3K/AKT/mTOR can regulate neuronal autophagy (47), but whether it regulates astrocyte autophagy in cerebral ischemia has not been determined. Beclin1, ATG7, and ATG16L are core autophagy regulatory factors, and miR-30a, β-arrestin 2, mir-379-5p, lncRNA snhg3, and lncRNA peg11as function as their upstream factors to regulate neuronal autophagy (97–100). Others, including autophagy-influencing factors transcription factor EB, syntaxin 17, and myotubularin-related protein 14 play specific roles in the autophagy of cerebral ischemic neurons (101–103). Interestingly, the downregulation of miR-30a in cerebral ischemic injury can enhance Beclin1-mediated neuronal autophagy to alleviate ischemic injury, and miR-30a also inhibits the expression of Beclin1 and autophagy in astrocytes. However, the effect of miR-30a on differences between astrocyte autophagy and neuron autophagy requires further investigation (66, 97, 98). HIF-1, UPR, neuron-specific protein conventional protein kinase Cγ (cPKCγ), and Shroom 4 are expressed in polarized cells such as neurons and can also regulate neuronal autophagy after cerebral ischemia (104–107). Despite numerous studies investigating differences in autophagy pathways between neurons and astrocytes after cerebral ischemia, the classic astrocyte autophagy pathway and the upstream regulator of autophagy core protein remain unclear. Few studies have investigated the differential regulation of autophagy in neurons and astrocytes under the same conditions. Such investigations may guide future research in the field of astrocyte autophagy.

## Crosstalk between astrocyte autophagy and other mechanisms in ischemic stroke

In the malignant secondary process of cerebral ischemic injury, astrocytes perform different functions at different stages, which are affected by many factors and mechanisms. Astrocytes activated by ischemic injury become reactive astrocytes, and under the influence of oxidative stress, inflammation, apoptosis, and other mechanisms caused by cerebral ischemic injury, they play a role in alleviating oxidative stress, releasing neurotrophic factors, producing excitatory toxicity, triggering inflammation, and glial scar formation (108). Astrocyte autophagy is one of the important regulatory mechanisms that plays an important role in the crosstalk between autophagy and other mechanisms.

### Oxidative stress

Many studies indicate that there is a close relationship between oxidative stress and autophagy. Specific manifestations of oxidative stress include excessive production of free radicals and oxidants or insufficient endogenous antioxidant capacity, resulting in damage to DNA, lipids, proteins, and other cellular components (109). The excessive production of reactive oxygen species (ROS) activates autophagy, resulting in the removal of irreversible oxidation molecules and the reduction of oxidative stress damage, whereas damaged or disordered autophagy leads to increased mitochondrial ROS under pathological conditions (25, 110). Astrocyte autophagy is activated in an H_2_O_2_ environment, and autophagy inhibitors can reduce H_2_O_2_-induced cell damage (111). Lead exposure induces autophagy and oxidative stress in astrocytes, and the use of autophagy inhibitors reduces oxidative stress levels (112). Hence, astrocyte autophagy may increase oxidative stress damage. After pretreatment with rapamycin and anthocyanin, autophagy levels and the viability of U87 cells were reportedly increased in an OGD environment, and anthocyanin had strong antioxidant activity and reduced cellular ROS levels (113). This suggests that induction of autophagy after cerebral ischemic injury may improve the capacity of astrocytes to resist oxidative stress (113). Whether astrocyte autophagy can exacerbate oxidative stress damage caused by cerebral ischemia is in urgent need of further research.

### Inflammation

As an intracellular protein degradation system, autophagy is involved in the regulation of inflammation. It eliminates inflammasome and pro-inflammatory cytokines, thereby limiting harmful and uncontrolled inflammation, whereas autophagy deficiency promotes the abnormal activation of inflammasomes, leading to many inflammatory diseases (109, 114). Rapamycin, an autophagy inducer, reportedly promotes the production of the astrocyte inflammatory factors TNF-α and iNOS in an OGD/R environment (69). Zha et al. (12) found that rapamycin-induced astrocyte autophagy downregulated the expression of inflammatory cytokines interleukin (IL) 1β and IL-6 in astrocytes under the influence of OGD/R, and the protective astrocyte autophagy induced may be offset by an inflammatory response with the prolongation of injury time. It can be inferred that the “struggle” between astrocyte autophagy and inflammatory responses also changes with the duration of ischemic injury, which further supports the contention that cerebral ischemia is affected by multiple complex mechanisms.

### Apoptosis and necroptosis

Under physiological and pathological conditions, autophagy and apoptosis—as two distinct manifestations of programmed cell death—have important roles in maintaining homeostasis. Autophagy interacts with apoptosis through complex mechanistic networks such as the interaction between autophagy-related proteins and caspases (115), and interactions between autophagy-related proteins Beclin1, Bcl-2, and Bcl-xL (116). Dynamic changes in autophagy and apoptosis within 7 days after stroke were assessed by determining the levels of LC3-II and cleaved caspase-3 following permanent MCAO injury (117). Results included activation and coexistence of autophagy and apoptosis within 12 h, as well as a marked decrease in the caspase-3 cleavage level and an increase in the LC3-II level after 4 days, suggesting a possible transition from apoptosis to autophagy, but it is unclear whether this transition occurred in neurons or glial cells (117). Another study indicated that autophagy may delay the initiation of apoptosis via the endogenous apoptotic pathway and protects astrocytes against OGD (67). After exposure to OGD combined with 3-MA for 1 h and 4 h, the levels of caspase-3 cleavage and caspase-9 were elevated in the astrocytes, and apoptosis was accelerated compared to control astrocytes. After 8 and 24 h of exposure, the levels of these factors were lower than those in control astrocytes (67).

Some progress has been made concerning the mutual regulatory mechanism of autophagy and apoptosis in astrocytes post-cerebral ischemia. Zhou et al. (63) reported that activation of the autophagy pathway after permanent MCAO can activate cathepsins B and L, cleave Bid, and elicit the translocation of cytochrome c from mitochondria to the cytoplasm, thereby activating caspase-3 in the ischemic cortex (63). *In vitro* experiments indicate that inhibition of astrocyte autophagy can block this process, which may be related to the reduction of lysosomal membrane instability caused by upregulation of heat shock protein 70.1B in astrocyte lysosomes (63). Under ischemic conditions, genetic knockout of ATG7 reduces caspase-3 and caspase-9 levels in astrocytes, whereas overexpression of miR-143-mediated TET1 induces 150-kDa oxygen-regulated protein, which in turn increases ATG7 levels via upregulation of glucose-regulated protein 78 (76). This contributes to the autophagy activation and apoptosis driven by caspase-3 and caspase-9 upregulation (76). Autophagy of OGD-induced astrocytes is suppressed and their apoptosis is increased after Beclin1 knockout (66). However, Beclin1 does not have a direct pro-apoptotic capacity, and its BH3 domain binds to members of the Bcl-2 family, thus playing a key regulatory role in autophagy and apoptosis (118, 119). c-Jun N-terminal kinase (JNK) can facilitate astrocyte apoptosis by increasing Bax/Bcl-2 levels after cerebral ischemic injury (120). Du et al. (121) reported that andrographolide accelerates autophagy and reduces the apoptosis of astrocytes after 12 h of hypoxia by activating the JNK pathway and regulating ATG5 in an *in vitro* astrocyte hypoxia injury model. Whether the JNK pathway affects the crosstalk between astrocyte autophagy and apoptosis in ischemic stroke warrants further investigation.

Astrocyte autophagy is closely associated with necroptosis. The regulatory or programmed form of necrosis is called necroptosis, of which receptor-interacting protein kinase (RIPK) is an important regulatory protein (122). Ryan et al. (123) investigated time-dependent changes in RIPK1, RIPK3, and apoptosis-related and autophagy-related proteins within 48 h after whole cerebral ischemia-reperfusion injury in rats. They reported that ischemic injury may activate autophagy-triggered necroptosis, and described a contrasting effect with caspase-3-dependent apoptosis (123). Another study indicated that RIPK1-mediated necroptosis may activate the autophagy-lysosome pathway by elevating LC3-II and active cathepsin B levels, thereby leading to ischemia-caused astrocyte death (124). In conclusion, autophagy, apoptosis, and necroptosis are modes of programmed cell death, and the relationship among autophagy, apoptosis, and necroptosis is not difficult to demonstrate, but the relevant mechanism has not been explained. Ferroptosis, a newly described type of autophagy-dependent programmed cell death type, is inextricably linked to autophagy. Unfortunately, to date, there is no reported study on the relationship between the two in astrocytes, which may be a new field that remains to be explored.

### Ubiquitin proteasome system

The ubiquitin proteasome system (UPS) is a non-lysosomal protein degradation system (125). It is capable of eliminating short-lived, damaged, and misfolded proteins (125). As a lysosomal protein degradation pathway, autophagy interacts with UPS (126). Inhibition of protease induces compensatory autophagy activation, and inhibition of autophagy promotes the accumulation of ubiquitinated substrates and their polymers (126). Previous studies confirmed that inhibition of proteasome upregulation of Beclin1 and LC3-I expression activated SHG-44 glioma cell autophagy (127). Further studies showed that when the proteasome is damaged, the astrocyte autophagy pathway is activated to resist proteasome stress damage (128). In a recent study, however, inhibition of UPS astrocyte autophagosomes increased slightly, accompanied by an increase in autophagy flux, and decreased with long-term inhibition of UPS astrocyte autophagy flux, which may be due to the inability of specific aggregates in astrocytes to degrade via the autophagy pathway (129). Notably, there is a shift from a UPS to an autophagy-mediated protein degradation pathway from ischemic injury to severe injury (52). Impaired UPS capacity in cerebral ischemia leads to the activation of compensatory astrocyte autophagy by massive fused in sarcoma (FUS) aggregate and may lead to excessive autophagy (130). Therefore, there are interactions and crosstalk between UPSs and autophagy in ischemic astrocytes, and how to use the mechanism and relationship to treat an ischemic injury is a new challenge.

## Astrocyte autophagy and neurons in ischemic stroke

Under physiological and pathological conditions, astrocytes and neurons are inseparable in terms of structure and function with respect to maintaining the functional stability and health of the central nervous system. Astrocytes are structurally closely related to neurons, with their fine branch processes linked to all parts of neurons (131). In the context of cerebral ischemia, astrocytes can supply metabolic substrates such as glucose and lactate, and release neurotrophic factors to protect neurons and maintain neuronal activity (131–133). Astrocytes can also secrete pro-inflammatory mediators that damage neurons (133). In an astrocyte-neuron co-culture model, OGD-induced autophagic flux in astrocytes enhances neuronal viability and attenuates apoptosis, whereas inhibition of autophagic flux counteracts this effect, which is consistent with *in vivo* experiments (11). Interestingly, the autophagy agonist rapamycin triggers autophagy in astrocytes after OGD/R, which can effectively reduce the release of IL-1β and IL-6 from astrocytes, contributing to a protective effect on neurons (12). G protein-coupled receptor 30 (GPR30) in astrocytes exerts a neuroprotective role in mouse MCAO, and activation of GPR30 restores astrocyte autophagy suppressed by glutamate and alleviates neuronal damage (134). In conclusion, astrocyte autophagy helps to protect neuronal activity after ischemia, and it plays a dual role after cerebral ischemic injury. Whether the injury of astrocyte autophagy affects neurons, and the specific mechanism of autophagy flux which plays a protective role in neurons, warrant further investigation.

## Astrocyte autophagy-targeted drug therapies for ischemic stroke

Therapeutic drugs targeting astrocyte autophagy can exert neuroprotective effects through different actions and pathways in ischemic stroke (Table 2). Rapamycin is an autophagy agonist that inhibits mTOR, initiating autophagy. It can induce autophagy in astrocytes after OGD/R, alleviating damage and reducing the infarct size in rats with transient MCAO (12). Similarly, dexmedetomidine upregulates autophagy in astrocytes by activating the TSC2/mTOR pathway and has a protective effect against cerebral ischemia (70). Ginkgolide K, a bioactive lactone extracted from *Ginkgo biloba* leaves, stimulates protective autophagy in astrocytes after OGD via the AMPK/mTOR/ULK1 pathway (72), and the *Salvia miltiorrhiz* extract salvianolic acid B exhibits a consistent effect (135). In contrast, *Dendrobium officinale* polysaccharides block AMPK/ULK1 pathway activation and have anti-autophagic effects on hypoxia/reoxygenation-stimulated astrocytes, promoting cell survival and reducing apoptosis (136). circAkap7-modified adipose-derived mesenchymal stem cell-sourced exosomes can target ATG12, facilitating astrocyte autophagy and ameliorating ischemic brain injury, and are thus of clinical therapeutic value (137). Other pharmacological studies suggest that both anthocyanins (113) and delta-opioid peptide [D-Ala2, D-leu5] enkephalin (138) confer protection against hypoxia-ischemia injury by inducing autophagy in astrocytes, whereas breviscapine (139), nimodipine (140), and propofol (141) have protective effects on hypoxia-ischemia injury via suppression of astrocyte autophagy. Many experimental studies have demonstrated that astrocyte autophagy is a potentially important therapeutic target in cerebral ischemia by utilizing the dual effects of astrocyte autophagy, but how to develop targeted drug therapy based on the characteristics of astrocyte autophagy requires further research.

**Table 2:** Astrocyte autophagy-targeted drug therapies for ischemic stroke.

**Compounds**	**Source**	**Effect on astrocyte autophagy**	**Therapeutic effects**	**References**
Anthocyanins	Polyphenol water-soluble pigment	Promotive	Promotion of cell viability by enhancing the autophagy in OGD-exposed U87 glioma cells	(113)
Breviscapine	A flavonoid derived from Erigerin breviscapus	Inhibitory	Neuroprotection through inhibition of astrocyte autophagy in MCAO	(139)
Delta Opioid Peptide [d-Ala2, d-Leu5] Enkephalin	Delta opioid receptors agonist	Promotive	Cytoprotective effects *via* inducing autophagy and inhibiting apoptosis in OGD-treated astrocytes	(138)
Dendrobium officinale polysaccharides	Main chemical constituents of Dendrobium candidum	Inhibitory	Promotion of cell viability by inhibiting H/R-induced activation of AMPK/ULK1 pathway and autophagy in astrocytes	(136)
Dexmedetomidine	A selective α2-adrenoceptor agonist	Promotive	Regulation of TSC2/mTOR triggers autophagy in astrocytes to alleviate OGD-induced cell damage	(70)
exo-circAkap7	Exosomes derived from circAkap7-modified adipose-derived mesenchymal stem cells	Promotive	Protection against ischemic injury through the promotion of ATG12-mediated autophagy in astrocytes under OGD/R	(137)
Ginkgolide K	A terpene lactone from Ginkgo biloba	Promotive	Pro-proliferative and pro-migratory effects through induction of astrocyte autophagy *via* the AMPK/mTOR/ULK1 pathway following OGD	(72)
Nimodipine	A 1,4-dihydropyridine L-type calcium channel antagonist	Inhibitory	Suppression of damage through inhibition of autophagy in OGD-exposed astrocytes	(140)
Propofol	A sedative agent	Inhibitory	Suppression of damage through inhibition of excessive autophagy in OGD-exposed astrocytes	(141)
Rapamycin	A natural compound	Promotive	Protection against OGD/R and I/R injury through induction of autophagy and suppression of inflammation in astrocytes *via* up-regulating Beclin-1 and LC3II and down-regulating IL-1β and IL-6	(12)
Salvianolic acid B	An active ingredient in Salvia miltiorrhiz	Promotive	Neuroprotective effect on cerebral ischemia by enhancing autophagy activity in astrocytes *via* the AMPK/mTOR/ULK1 pathway activation	(135)

Ref, references; OGD, oxygen-glucose deprivation; MCAO, middle cerebral artery occlusion; OGD/R, oxygen-glucose deprivation/reoxygenation; I/R, ischemia-reperfusion.

## Conclusions and perspectives

**Figure 3:**
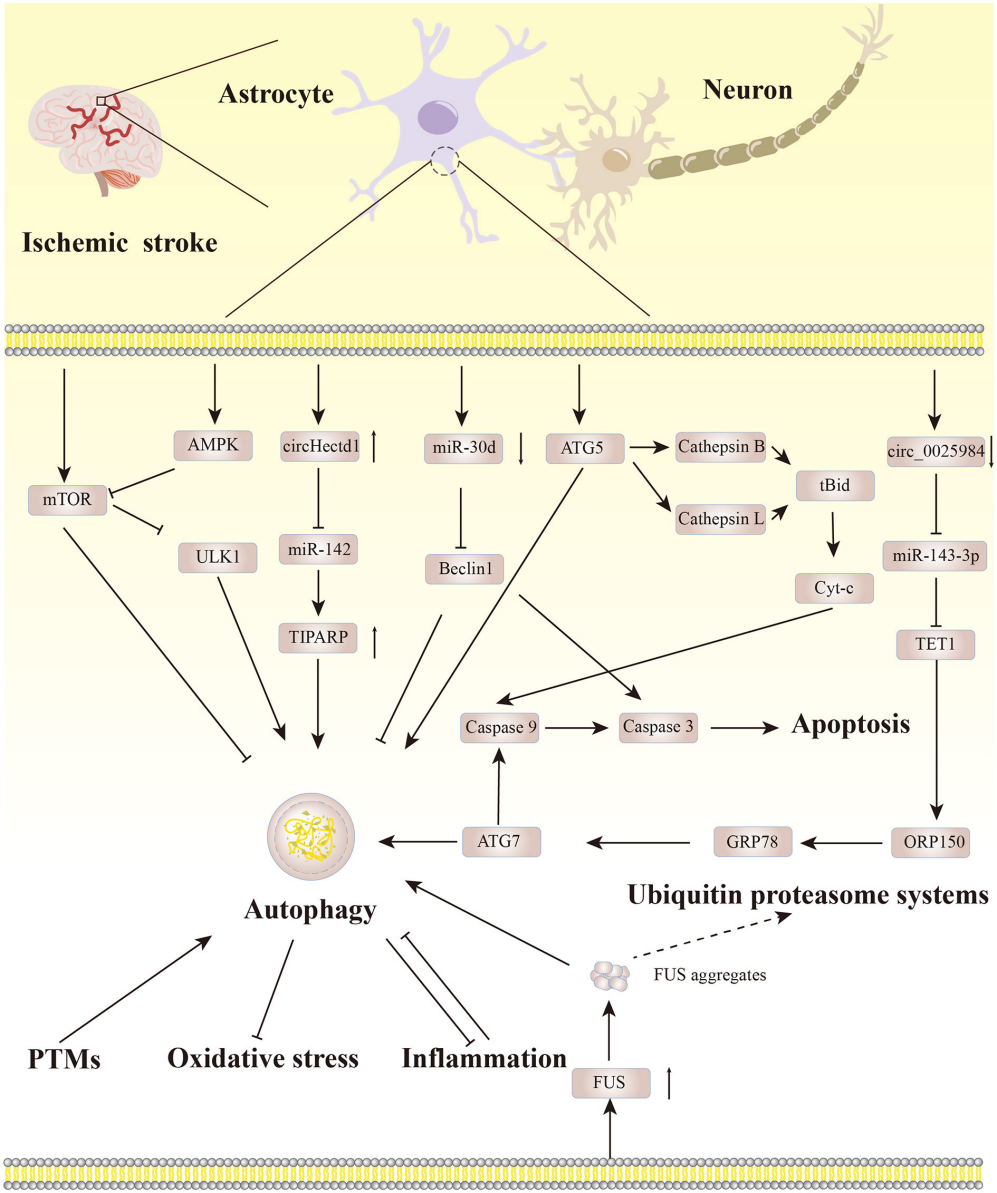
The regulatory pathways involved in astrocyte autophagy in ischemic stroke.

As a regulatory mechanism to maintain the protein balance in the body, autophagy is indispensable. Astrocyte autophagy becomes a “double-edged sword” in ischemic stroke, and there are crosstalks between astrocyte autophagy and other pathological mechanisms. Notably, targeting autophagic flux in astrocytes can reinforce the neuroprotective effect of astrocytes. Therefore, it is particularly important to control the level of autophagy in astrocytes. Based on the existing literature, we have summarized the regulatory pathways involved in astrocyte autophagy in ischemic stroke, as depicted in Figure 3.

With continued research, astrocyte autophagy may become a therapeutic drug target in cerebral ischemia, but the clinical efficacy of this approach remains to be validated. Notably, autophagy-related genes may have various effects on cells. For example, the autophagy-associated protein Beclin1 affects the autophagy process, but it also participates in the regulation of cell functions such as endocytic transport and LC3-related phagocytosis (119). Thus, there is an urgent need to develop therapeutic drugs targeting the regulation of astrocyte autophagy in ischemic stroke, which is conducive to elucidating the regulatory mechanisms of astrocyte autophagy and provides new directions and strategies for the clinical treatment of cerebral ischemia.

## Author contributions

P-WS: wrote the manuscript and draw the pictures. ZZ, TW, Y-NZ, YW, KM, B-BH, Z-CW, and H-YY: proofread the manuscript. H-JZ and S-JW: reviewed and revised the manuscript. All authors contributed to this article and approved the submitted version.

## Funding

This work was supported by a grant from the National Natural Science Foundation of China (81874411), the Joint Foundation for Innovation and Development of the Natural Science Foundation of Shandong Province (ZR2021LZY014), the Shandong Province Universities' Development Plan for Youth Innovation Teams (2019-9-201, 2019-9-202, and 2019KJK013), and the Natural Science Foundation of Shandong Province (ZR2019ZD23).

## Conflict of interest

The authors declare that the research was conducted in the absence of any commercial or financial relationships that could be construed as a potential conflict of interest.

## Publisher's note

All claims expressed in this article are solely those of the authors and do not necessarily represent those of their affiliated organizations, or those of the publisher, the editors and the reviewers. Any product that may be evaluated in this article, or claim that may be made by its manufacturer, is not guaranteed or endorsed by the publisher.
